# Management of Arachnoid Cysts: A Comprehensive Review

**DOI:** 10.7759/cureus.2458

**Published:** 2018-04-10

**Authors:** Fatima Mustansir, Sanaullah Bashir, Aneela Darbar

**Affiliations:** 1 Surgery, The Aga Khan University

**Keywords:** arachnoid cyst, neuroendoscopy, microsurgical fenestration, cystoperitoneal shunting, suprasellar cyst, intrahemispheric cyst, middle fossa cyst, arachnoid cyst, quadrigeminal cyst, neuroendoscopy, microsurgical fenestration

## Abstract

Arachnoid cysts are non-neoplastic, intracranial cerebrospinal fluid (CSF)-filled spaces lined with arachnoid membranes. Large arachnoid cysts are often symptomatic because they compress surrounding structures; therefore, they must be treated surgically. As several surgical management options exist, we explore the best approach according to each major type of arachnoid cyst: middle cranial fossa cyst, suprasellar cyst, intrahemispheric cyst, and quadrigeminal cyst.

## Introduction and background

Arachnoid cysts can be classified as primary developmental cysts or secondary cysts. Primary cysts arise from the splitting of the arachnoid membranes in utero, resulting in the development of anomalous collections of cerebrospinal fluid (CSF). Secondary cysts are less common, often appearing after trauma, surgery, infection, or intracranial hemorrhage. Arachnoid cysts comprise 1% of all intracranial space-occupying lesions [1]. The prevalence in adults is approximately 1.4% with a female preponderance, while the prevalence in children is 2.6% [2-4].

The signs and symptoms of arachnoid cysts vary according to their size and location. Small cysts are usually symptomatic, requiring observation and follow up. However, larger cysts can have a mass effect on neurovascular structures, leading to neurological symptoms [5]. Headaches are the most common symptom, accounting for a share of 66% [6]. Other symptoms include dizziness, nausea, vomiting, worsening of mood, mental status changes, ataxia, seizures, and hearing loss [7].

While arachnoid cysts vary in their location, most are supratentorial and found in the middle fossa. The remainder may occur in the cerebellopontine angle, suprasellar and quadrigeminal cisterns, cerebral convexities, and cisterna magna [2, 8].

**Figure 1:**
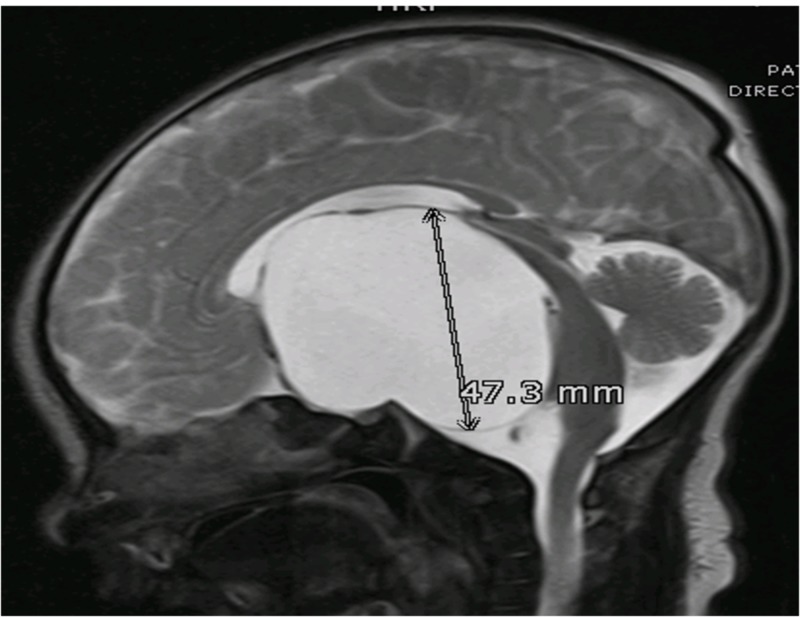
MRI T2WI, Sagittal Section A large extra-axial, well-defined CSF intensity cystic lesion is identified in the midline in the sellar and suprasellar region. It measures 48 x 62 x 47 mm in AP transverse and cranio-caudal dimension. This lesion is causing a compressive effect on the third ventricle and bilateral lateral ventricles. These findings are consistent with arachnoid cyst. MRI T2WI: magnetic resonance imaging T2 weighted image; CSF: cerebrospinal fluid.

Due to the possibility of compression of neurovascular structures by large arachnoid cysts (Figure 1), a surgical approach is preferable to passive observation, as is done with smaller cysts.

With regards to large arachnoid cysts, there has been no consensus on the single best management strategy. The most frequently used methods for treating arachnoid cysts are microsurgical fenestration via craniotomy, neuroendoscopic fenestration (Videos 1, 2) and cystoperitoneal shunting [5, 9-10].

**Video 1:** Endoscopic fenestration of arachnoid cyst: Part 1

**Video 2:** Endoscopic fenestration of arachnoid cyst: Part 2

Choi et al. propose three categories of cysts on the basis of their clinical presentation and reasons for surgery [11]. In the first group, the cysts presented with symptoms of hydrocephalus and intracranial hypertension. In the second group, the cysts presented with vague symptoms of dizziness, headaches, large head, skull abnormalities, strabismus, seizures, and developmental delays. There were minimal or no clinical symptoms in the third group, but the patients were surgically treated anyway, in expectation of improvement of abnormal radiological and neuropsychological ﬁndings. They found that only the first group had a reasonable improvement rate, while there was a partial improvement in the third group, and minimal improvement in the second group. Therefore, surgical evaluation is advocated only in those patients whose symptoms are directly related to the cyst.

Neuroradiological imaging is used to make a choice between neuroendoscopy and shunting. With the neuroendoscopic approach, imaging is used to identify an area of contiguity between the ventricular ependyma and the wall of the cyst; this is opened via an endoscope to facilitate continuous drainage of the cyst. A minimum opening of 10–15 mm has to made, along with the removal of the cyst wall, in order to prevent the closure of the stoma again [9].

The efficacy of the endoscopic approach with arachnoid cyst fenestration is still a topic of debate. Several studies have been done to evaluate its importance. A study done by Jung Won Choi et al. reviewed the surgical outcomes of intracranial arachnoid cysts and concluded that fenestration of cysts is associated with more surgical complications [11]. However, some studies suggest that neuroendoscopy remains a superior approach, with a lower risk of surgical complications associated with craniotomy and shunting [12-13]. In a case series on the endoscopic approach, a success rate of up to 71–81% has been documented [12]. Furthermore, with associated hydrocephalus, the possibility of larger spaces makes this treatment option easier to execute.

The location of the arachnoid cyst also determines the surgical approach and outcome. In terms of endoscopy, the best results have been reported with suprasellar cysts. In patients with middle fossa cysts, endoscopy remains controversial; some authors favor microsurgery over endoscopic surgery for cysts in this location [14]. Furthermore, treating large hemispheric arachnoid cysts in infants using endoscopic fenestrations have had less favorable outcomes than the same treatment in older children. However, a neuroendoscopic approach is still preferred over shunting in this population too, owing to the high risk of shunt failure [15].

## Review

Middle cranial fossa arachnoid cyst

Approximately 50-65% of arachnoid cysts occur in the middle cranial fossa [8].

The Galassi classiﬁcation is used to classify middle fossa cysts into three types [16]. Type I cysts are typically asymptomatic and are present in the anterior middle cranial fossa. Type II cysts extend superiorly along the Sylvian ﬁssure, occasionally displacing the temporal lobe. Finally, Type III cysts are very large and occupy the entire middle cranial fossa, displacing not only the temporal lobe but also disrupting the parietal and frontal lobes.

Type I cysts are best treated by microsurgical fenestration. The latter two are accessed via endoscope. The appearance of the chiasmatic and interpeduncular cisterns on magnetic resonance imaging (MRI) helps to decide between endoscopic and microsurgical fenestration. Endoscopic cystocisternotomy is advocated when there is ample space between the third cranial nerve and the tentorial notch and between the optic nerve and the carotid artery within large cisterns with thin membranes, while microsurgical techniques remain a suitable option in cases of fenestration of deep thicker membranes in the vicinity of vital structures [17].

Various factors have to be considered before performing endoscopic fenestrations into the basal cisterns. The medial wall of the cyst is coursed by the arterial vessels of the Sylvian fissure that can be damaged when stoma is enlarged with sharp instruments. Moreover, the membranes of these cysts are difficult to penetrate due to their rich collagen content, so a sharp instrument or scissors are often used [18].

The literature related to middle fossa cyst treatment is not as diverse or as reliable as it is for other types of cysts. A recent meta-analysis concluded that while all three surgical methods (endoscopic, microsurgical, and shunting) are effective for the management of middle fossa cysts, endoscopic fenestration is the preferred primary surgical modality. The latter two options should only be considered when symptoms are unchanged after endoscopic treatment [19].

Suprasellar arachnoid cyst

Suprasellar arachnoid cysts are usually found in close proximation to the third ventricle. They present with hydrocephalus.

Endoscopic fenestration of such cysts is the standard modality of treatment [20]. Open procedures are usually avoided due to a significantly higher morbidity, and the fact that the success rate does not go above 70%. When performing endoscopic fenestration, surgeons adopt different techniques. These range from fenestrating only the apical membrane, usually at the level of the foramen of Monroe, between the ventricle and the cyst (ventriculocystostomy), to basilar fenestration toward the prepontine cistern (cyst-cisternostomy), called ventriculocystocisternostomy (VCC).

A study conducted using MR-imaged CSF ﬂow dynamics shows that fenestration of suprasellar cysts should be done in both the ventricles and in the basal cisterns, to prevent relapse of symptoms [21]. In a paper on the management of suprasellar arachnoid cysts, Gui et al. concluded that endoscopic ventriculocystocisternostomy is far more effective than ventriculocystostomy [20].

Interhemispheric arachnoid cyst

Mori et al. classified inter-hemispheric cysts into two types: parasagittal and midline [22].

Parasagittal cysts are unilateral; they are found in toddlers and are infrequently associated with agenesis of the corpus callosum. As these cysts are not near the ventricles, they do not usually present with hydrocephalus. Hence, the best treatment is excision of the cyst along with its lining. Midline cysts are complex, multiloculated cysts usually discovered at birth and associated with agenesis of the corpus callosum. They do present with hydrocephalus. The most frequent site of contiguity between the cyst and the ventricles is at the level of the roof of the third ventricle. Therefore, fenestration of the cyst into the third ventricle is required. Moreover, the septations have to be broken and made to communicate with the nearby cistern [23-24].

Quadrigeminal arachnoid cyst

Quadrigeminal cistern cysts are uncommon; only 79 cases were reported in the literature up to 2008 [25].

Quadrigeminal cysts may be classified into three types. Type I are cysts with supratentorial and infratentorial extension, Type II are cysts with infratentorial extension (supracerebellar or supra-retrocerebellar), and Type III includes cysts with lateral extension toward the temporal lobe. As quadrigeminal arachnoid cysts compress or distort the cerebral aqueduct at an early stage, they are usually associated with hydrocephalus when symptomatic. Symptoms include macrocrania, headaches, vomiting, lethargy, papilledema, and impairment of upward gaze and other ocular disorders [26]. Due to the compressive symptoms produced by these cysts, it is imperative that they be treated.

Minimally invasive treatment of these cysts is preferred due to their precarious proximity to the pineal quadrigeminal region. The endoscopic technique used varies according to the extension of the cyst. It can extend to the trigone superiorly, to the supracerebellar cistern inferiorly, and to the third ventricle anteriorly. Further endoscopic approaches are cyst fenestration and removal via the suboccipital supracerebellar approach, lateral ventriclecystostomy, and third ventriclecystostomy [27].

Two studies that analyzed 14 and 18 cases of quadrigeminal cistern arachnoid cysts treated with the above mentioned endoscopic approaches, found that patients were shunt independent in 78% and 93% of the cases respectively [26, 28]. Cinalli et al. concluded that arachnoid cysts of the quadrigeminal cistern can be effectively treated by endoscopy, with a success rate of 90% observed in the series if endoscopy was the first line of treatment. Specifically, they stated that endoscopic third ventriculostomy should be combined with ventriculocystostomy to offer the highest success rate with a single procedure [26].

## Conclusions

As mentioned earlier, the previous techniques used to manage arachnoid cysts were craniotomy and marsupialization of the cyst or insertion of a cystoperitoneal shunt. While these techniques still remain useful, neurosurgeons are increasing turning to endoscopic means of management. The endoscopic management of intracranial arachnoid cysts is a safe and effective therapeutic modality that results in a high success rate. The approach, trajectory, and site of fenestration must be planned individually in each case by using preoperative MR imaging and must be studied carefully intraoperatively. However, the cyst location is important for surgical decision-making. Arachnoid cysts in the suprasellar and quadrigeminal regions are most amenable to neuroendoscopy. On the other hand, interhemispheric cysts should be treated by microsurgical fenestration.
